# Study on the mechanism of action of the active ingredient of Calculus Bovis in the treatment of sepsis by integrating single-cell sequencing and machine learning

**DOI:** 10.1097/MD.0000000000042184

**Published:** 2025-04-18

**Authors:** Hao Wang, Yingchun Hu, Li Hu

**Affiliations:** a School of Clinical Medicine, Shandong Second Medical University, Weifang, People’s Republic of China; b Department of Emergency Medicine, The Affiliated Hospital of Southwest Medical University, Luzhou, People’s Republic of China.

**Keywords:** calculus bovis, machine learning, molecular docking, molecular dynamics simulation, sepsis, single-cell RNA-seq

## Abstract

**Background:**

Sepsis, a complex inflammatory condition with high mortality rates, lacks effective treatments. This study explores the therapeutic mechanisms of Calculus Bovis in sepsis using network pharmacology and RNA sequencing.

**Methods:**

Sepsis data from the China National GeneBank Database were analyzed for differentially expressed genes (FC ≥ 2, FDR < 0.05). Active components of Calculus Bovis were identified via the HERB and BATMAN-TCM databases, with target interactions assessed through protein-protein interaction (PPI) networks. GO and KEGG analyses identified pathway enrichments (*P* ≤ .01). Survival analysis using the GSE65682 database evaluated prognosis-related genes (*P* < .05). Four machine learning models (XGBoost, SVM, Decision Tree, KNN) were constructed to assess diagnostic potential, with AUC values evaluating accuracy. Immunofluorescence and single-cell RNA sequencing localized key genes, while molecular docking and molecular dynamics simulations (MD) assessed binding affinities and stability of Calculus Bovis compounds with target proteins.

**Results:**

We identified 593 targets for Calculus Bovis and 4329 sepsis-related genes, with 149 overlapping. Key genes ADAM17, CASP1, CD81, and MGMT were linked to improved prognosis (*P* < .05) and involved in inflammatory responses and pyroptosis (*P* ≤ .01). The XGBoost model achieved high diagnostic accuracy (AUC: training = 1.000, test = 0.964). Molecular docking showed strong binding (energy < −6.0 kcal/mol), and MD indicated stable interactions, particularly with ADAM17 and CD81.

**Conclusion:**

This study highlights the potential of Calculus Bovis in sepsis treatment, identifying key genes as therapeutic targets.

## 1. Introduction

Sepsis can be defined as a systemic inflammatory response syndrome triggered by the infiltration of harmful microorganisms, including bacteria. Septic shock, its most severe form, is characterized by a drop in blood pressure, decreased tissue perfusion pressure, and hypoxia indicative of shock. Globally, sepsis continues to be a major cause of mortality.^[1]^ The organ damage in sepsis involves multiple systems, and the underlying mechanisms are complex, without specific and effective treatment currently available. Given these circumstances, developing effective drugs for the treatment of sepsis is crucial. “Angong Niuhuang Wan,” first recorded in the Qing dynasty’s traditional Chinese medicine (TCM) compendium “Wen Bing Tiao Bian,” has been long used clinically for treating pyrexia, febrile convulsions, encephalitis, and sepsis, owing to its anti-inflammatory, antioxidant, anti-cell-death, anti-convulsant, anti-edema, antipyretic, antithrombotic, neuroprotective, and cardiovascular protective effects.^[2]^ Calculus Bovis, the sovereign medicine in Angong Niuhuang Wan, is a traditional animal-based medicine widely used for treating various diseases, including high fever, convulsions, and stroke.^[3]^ Its chief components include isoiridogermanal, ergotamine, and ursolic acid. Modern medical research indicates that Calculus Bovis exerts antioxidant, anti-inflammatory, and anti-apoptotic effects.^[4]^

However, the potential mechanisms whereby Calculus Bovis improves the prognosis of sepsis are incompletely understood. Network pharmacology, a new field focused on building multi-dimensional networks that interconnect diseases, phenotypic presentations, genes, and drug interactions, is dedicated to the holistic prediction of drug targets and enhancing the efficacy of drug development processes.^[5]^ The holistic perspective of TCM aligns well with the network and systems approach of network pharmacological research.^[6]^ This research was conducted to uncover the potential targets and mechanisms whereby Calculus Bovis operates in sepsis therapy. To that end, network pharmacology and RNA sequencing (RNA-seq) techniques were employed, along with widely used public databases. Table 1 shows the abbreviations for the entire study.

**Table 1:** List of abbreviations

Abbreviation	Full name
MD	molecular dynamics simulations
TCM	traditional Chinese medicine
RNA-seq	RNA sequencing
CNGB	China National GeneBank Database
SCCM	Society of Critical Care Medicine
ESICM	European Society of Intensive Care Medicine
PCA	principal component analysis
FC	fold change
FDR	false discovery rate
PPI	Protein-protein interaction
SVM	Support Vector Machine
DT	Decision Tree
KNN	K Nearest Neighbor
HPA	Human Protein Atlas

## 2. Methods

### 2.1. Data sources

The original sepsis data were sourced from the China National GeneBank Database [https://db.cngb.org/] under accession number CNP0002611. This dataset consists of venous blood samples collected from 23 hospitalized sepsis patients in the intensive care unit of the Emergency Department at Southwest Medical University Affiliated Hospital from February 2019 to December 2020. Additionally, venous blood samples from 10 healthy volunteers were collected as a control group. This dataset conforms to the Sepsis 3.0 criteria jointly published by the Society of Critical Care Medicine and the European Society of Intensive Care Medicine in 2016, which includes infection and a change in the SOFA score of ≥ 2.

### 2.2. Selection of differentially expressed RNA

The data were analyzed using the online tool iDEP96^[7]^ (http://bioinformatics.sdstate.edu/idep96/) for box plot, volcano plot, and principal component analysis (PCA). Box plots were used to ensure the comparability and reliability of the dataset. PCA was employed to identify and exclude outlier samples. Volcano plots were utilized to visually identify significantly upregulated and downregulated differential genes. Subsequently, differential expression analysis was conducted using the DESeq2 method, setting a minimum fold change of 2 and a false discovery rate below 0.05 to identify differentially expressed RNA.

### 2.3. Selection of active components and targets of *Calculus Bovis*

The HERB database^[8]^ (http://herb.ac.cn/) functions as a comprehensive high-throughput experimental and reference resource specifically for TCM, integrating various data resources and providing comprehensive lists of herbs, components, targets, and diseases. Active components of *Calculus Bovis* were obtained from the HERB database by using a molecular weight of less than 500 as a screening criterion. Target prediction for these active components was performed using the SwissTargetPrediction database^[9]^ (http://www.swisstargetprediction.ch/). Furthermore, active components and targets of *Calculus Bovis* were identified from the BATMAN-TCM database^[10]^ (http://bionet.ncpsb.org.cn/batman-tcm/index.php/), with selection criteria of component fraction greater than 20 and *P*-value less than 0.05. The components and targets obtained from both databases were then cross-referenced and deduplicated. After removing duplicate targets of *Calculus Bovis* and sepsis disease targets, the intersecting genes were extracted, and a Venn diagram (http://www.liuxiaoyuyuan.cn/) was created to display these intersecting genes.

### 2.4. PPI analysis

Protein-protein interaction (PPI) networks aid in understanding the interactions of proteins within cells, thereby revealing the mechanisms and regulatory modes of biological processes and further screening potential key targets. The STRING database^[11]^ (www.string-db.org) is a repository for PPI networks, aggregating data from public databases and published literature. The organism chosen for the study was *Homo sapiens*. A minimum interaction threshold of 0.4 was established, and opted to conceal nodes that were not connected. Subsequently, the 149 genes that intersected were uploaded into the network platform to build the PPI network.

### 2.5. Construction of the “Active Component–Target–Drug” network

To further clarify the mechanisms of *Calculus Bovis* in treating sepsis, a network graph of active component targets for the treatment of sepsis was established using Cytoscape 3.10.1, with degree analysis performed using its plugin, Network Analyzer. The size of the network nodes is positively correlated with their degree. The more connections a node has, the higher its degree value.

### 2.6. GO and KEGG enrichment analysis (METASCAPE)

Metascape^[12]^ (http://metascape.org/), a gene function annotation analysis tool, assists users in applying bioinformatics analysis methods to batch genes and protein analyses, facilitating understanding of gene or protein functions. GO annotation is divided into 3 parts for comprehensive analysis: biological processes, cellular components, and molecular functions. KEGG includes gene pathway information for different species. Filtering was conducted using *P* ≤ .01, and visualization of the results was performed using the Oebiotech platform (https://cloud.oebiotech.com/).

### 2.7. Survival curve analysis

Survival curve analysis is primarily used to analyze the clinical significance of key targets. To explore whether the key genes identified in this study have the potential to determine prognosis, the public database GSE65682^[13]^ was downloaded. This data comprised peripheral blood specimens from 478 patients diagnosed with sepsis, inclusive of their gene expression profiles and clinical prognostic data for 28 days. Gene expression levels were ordered in a descending sequence. The upper 50% of the dataset (comprising 293 genes) was classified as the low-expression group, while the lower 50% was defined as the high-expression group. A *P* value of no more than .05 was considered as proof of statistical significance in the log-rank test used for statistical analysis.

### 2.8. Construction of the diagnostic model

In this study, we constructed a diagnostic model for sepsis by 4 machine learning algorithms (XGBoost, Support Vector Machine (SVM), Decision Tree, and K Nearest Neighbor (KNN)) using the R language 4.4.1. The data were derived from the GSE65682 dataset, and all the data were standardized and divided into a training set and a test set according to the ratio of 7:3, which ensured that the model performance was robust.XGBoost: XGBoost is a decision tree integration algorithm based on gradient boosting, which can effectively handle large-scale, high-dimensional data.^[14]^ We set objective = “binary: logistic” (binary classification task), eval_metric = “auc” (AUC as the evaluation criterion), max_ depth = 6 (maximum depth of the tree), and eta = 0.3 (learning rate) are parameters for model training. SVM: the SVM is an algorithm that achieves classification by finding the optimal hyperplane for high-dimensional feature spaces.^[15]^ In this study, a radial basis function (RBF kernel) was used as the kernel function, and the data was normalized. The SVM model uses the probability = TRUE parameter to output the classification probability of the sample. Decision Tree: A decision tree is a classification algorithm that recursively divides a dataset into subsets.^[16]^ In this study, the decision tree model was trained using the method = “class” parameter, and its predictive performance was evaluated by the probability values output by the model. KNN: KNN is a non-parametric classification algorithm based on the distance.^[17]^ In the study, k = 5 was used as the nearest neighbor number, and the data were normalized to ensure the consistency of the magnitude between different features. In addition, SHAP (Shapley Additive exPlanations) values were used to interpret the feature importance of the XGBoost model. We used the Shapviz package to generate feature importance plots to quantitatively demonstrate the impact of each gene on the final prediction results.

### 2.9. Immunofluorescence analysis

In this study, data from the Human Protein Atlas (HPA) database (https://www.proteinatlas.org/) were utilized for immunofluorescence analysis and tissue-specific expression analysis for target genes.^[18]^ The HPA database is a publicly available biological database covering protein expression data from the human genome, providing extensive protein localization and tissue expression information. Immunofluorescence analysis: we obtained immunofluorescence images of ADAM17, MGMT, CD81, and CASP1 from the HPA database to demonstrate the subcellular localization characteristics of these target proteins. Green fluorescence in the immunofluorescence images represents the expression of target proteins, red fluorescence labels microtubules, and blue staining is used to show the nuclei. Tissue-specific expression analysis: In this study, the expression data of these 4 genes in several normal human tissues were also obtained from the HPA database. The mRNA expression levels of each gene in different tissues were quantified by nTPM (normalized Transcripts Per Million) values. This data, derived from RNA sequencing analysis, can reveal the specific expression patterns of the target genes in multiple tissues, thus supporting further exploration of the biological functions of these genes.

### 2.10. Single-cell sequencing

To investigate the cellular lineage localization of key genes in peripheral blood mononuclear cells, we collected 5 PBMC samples for 10x single-cell RNA-seq. The samples included those from 2 individuals in good health, a patient experiencing systemic inflammatory response syndrome, and 2 individuals diagnosed with sepsis. Quality control for these datasets was conducted via the Cell Ranger online platform. This platform incorporates the STAR14 software, which aligns the read data to the reference genome. Single-cell transcriptomic sequencing was performed by utilizing a combination of transcript sequences, unique molecular identifiers, and cell barcodes. This approach was adopted to acquire precise counts of individual transcript molecules in each cell. In this project, the employed dimensionality reduction techniques included the mutual nearest neighbors algorithm and the t-distributed stochastic neighbor embedding method. Results from dimensionality reduction utilizing the MNN approach were analyzed through t-SNE visualization, which facilitated the identification of ideal cell populations. Marker genes are those that are expressed marginally in other populations but significantly in the majority of cells within a particular cell population were significantly upregulated in their respective cell groups. Selected marker genes were used to identify cell populations by constructing a single-cell library related to sepsis. This analysis involved transcriptomic sequencing of 5 cell samples. The outcomes of the analysis indicated that macrophages were characterized by groups 3 and 5, natural killer cells by group 4, T cells by groups 1, 2, 6, and 8, B cells by group 7, and platelets by group 9.^[19]^

### 2.11. Molecular docking

Following the establishment of the network, PPI analysis, and survival curve analysis, we selected key proteins and their corresponding active components for molecular docking. In this research, AutoDock Vina 1.4.2, a specialized computational tool for protein-ligand docking, was used to assess the binding energies and modes of interaction between potential drugs or small molecules and their respective targets. The three-dimensional conformations of these drugs or small molecules were obtained from the PubChem database^[20]^ (https://pubchem.ncbi.nlm.nih.gov/), while the protein structures were sourced from the PDB database^[21]^ (https://cn.string-db.org/). Initially, both the protein and ligand files were prepared and converted into the PDBQT format, which included the removal of all water molecules and the addition of polar hydrogen atoms and Kollman charges.^[22,23]^ Each protein structure domain was surrounded by a grid box, allowing the molecules to move freely. The docking pocket was characterized by a cubic pocket measuring 30 A × 30 A × 30 A, with a grid point spacing of 0.05 nm.

### 2.12. Molecular dynamics simulation

Molecular dynamics simulations (MD) are performed based on the initial docking structure of protein-small molecule complexes with the aim of investigating the stability and interactions between target proteins and small molecule ligands under dynamic conditions. In this study, 100 ns all-atom MD were performed using GROMACS 2020.4 software.^[24]^ The proteins were treated with the AMBER ff14SB force field, and the small molecules with the GAFF force field. Small molecule charges were obtained by calculations using the Hartree-Fock (HF) SCF/6-31G* method with the quantum chemistry software Gaussian 09. The system was solvated with the addition of the TIP3P water model to form an octahedral water box with a truncation distance of 10 Å. The charge of the system was balanced by Na + or Cl- ions. During the simulation, the whole system was first energy-optimized, including the 2500-step steepest descent method and the 2500-step conjugate gradient method. Next, a 200 ps NVT tethered simulation was used for warming, which gradually increased the system from 0 K to 298.15 K. Subsequently, a 500 ps NPT tethered simulation was performed to equilibrate the system, maintaining a pressure of 1 atm during this process. Finally, production simulations under NPT conditions were performed for 100 ns, using the particle mesh Ewald method to calculate long-range electrostatic interactions and the SHAKE method to constrain hydrogen-containing bond lengths. Trajectories were saved every 10 ps for subsequent analysis. During the simulations, we calculated and analyzed the solvent accessible surface area, radius of gyration, root-mean-square deviation, and root-mean-square fluctuation of each protein-small-molecule complex in order to assess the stability of the complexes and conformational changes.

## 3. Results

### 3.1. Differential screening results

**Figure 1.**
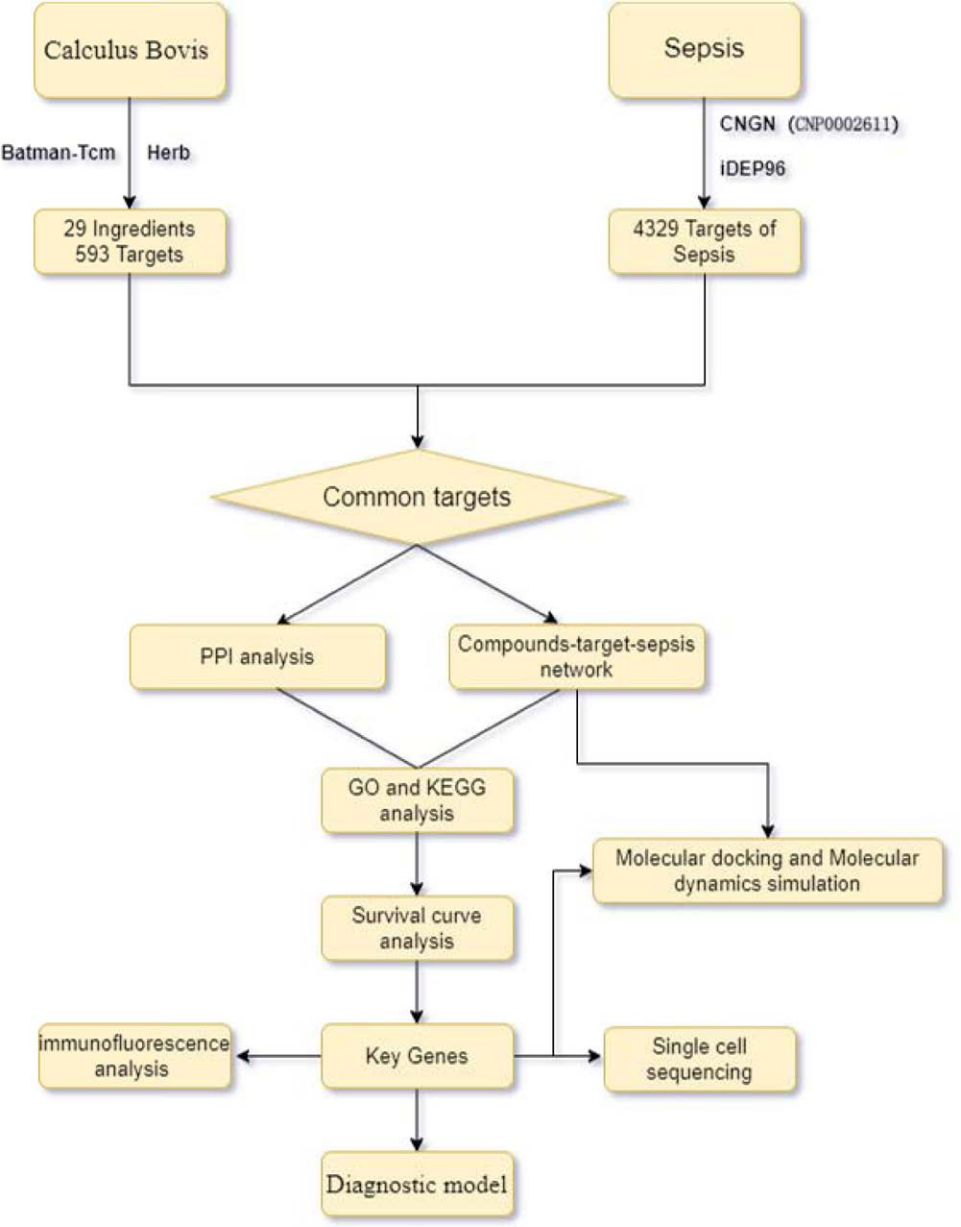
Study Flowchart. Investigating the prospective active components and underlying mechanisms of Calculus Bovis in the management of sepsis through network pharmacology and RNA sequencing techniques.

**Figure 2.**
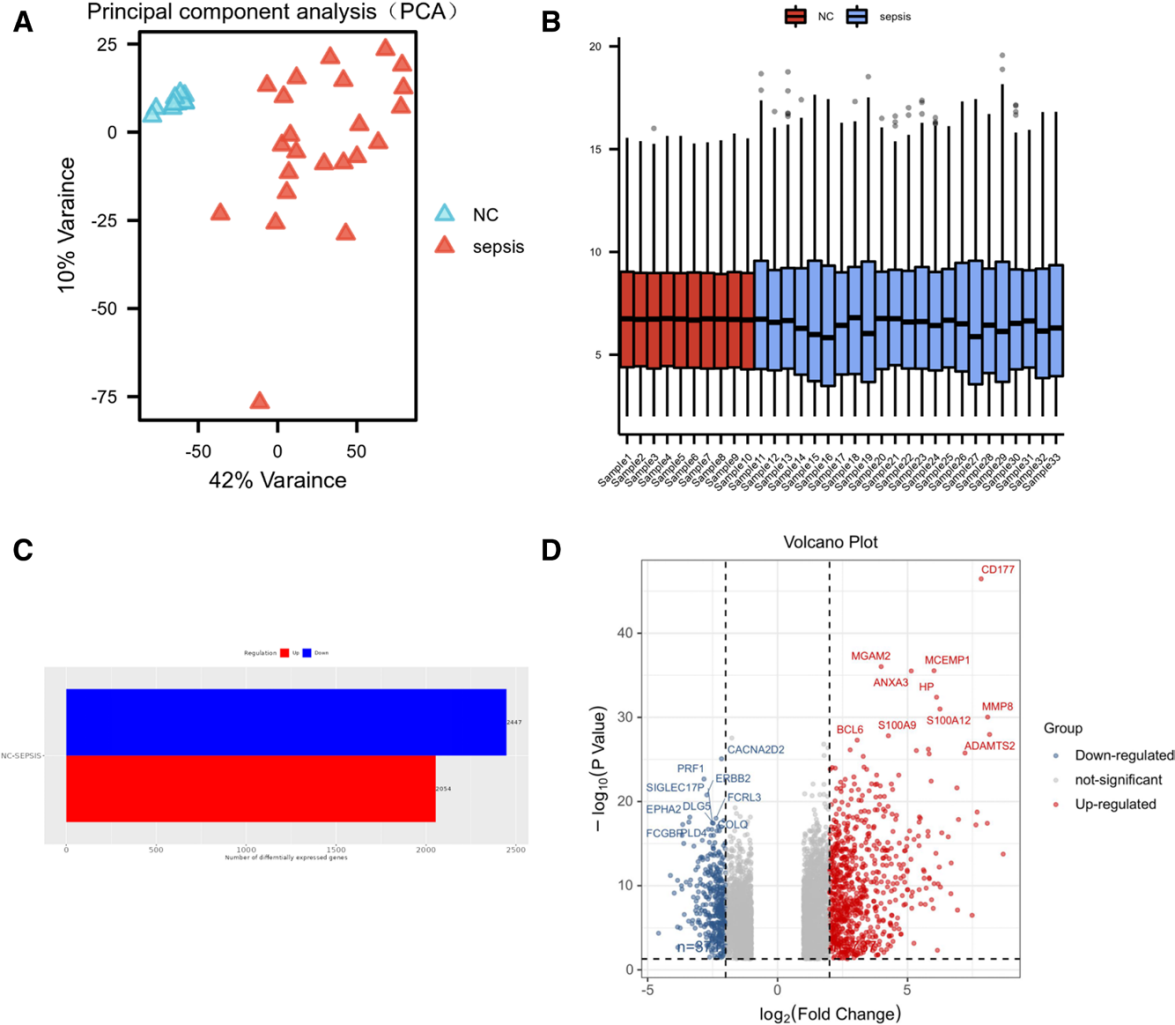
Quality Control and Screening of Differentially Expressed Genes. A. PCA demonstrates significant distinctions between the experimental and control groups, excluding outlier samples. B. Box plots show data evenly distributed at the same level across each sample, ensuring comparability. C. Genes that are elevated (red) and downregulated (blue) in histograms. In the graph, as the vertical axis represents the distinct groups, the horizontal axis represents the number of distinctly expressed RNAs. D. The blue and red colors in the volcano plot represent down-regulated and up-regulated genes, respectively.

The flow chart of the study is shown in Figure 1. The dataset was made to undergo box plot analysis, volcano plot analysis, and PCA to ensure its reliability and comparability. A total of 4501 genes exhibited differential expression (Fig. 2A–D). In the group with sepsis, there were 2447 RNAs marked by elevated expression and 2054 RNAs characterized by reduced expression. Upon exclusion of duplicates and nonstandard genes, 4329 genes associated with the disease were identified.

### 3.2. Selection of active components and targets of calculus bovis

**Figure 3.**
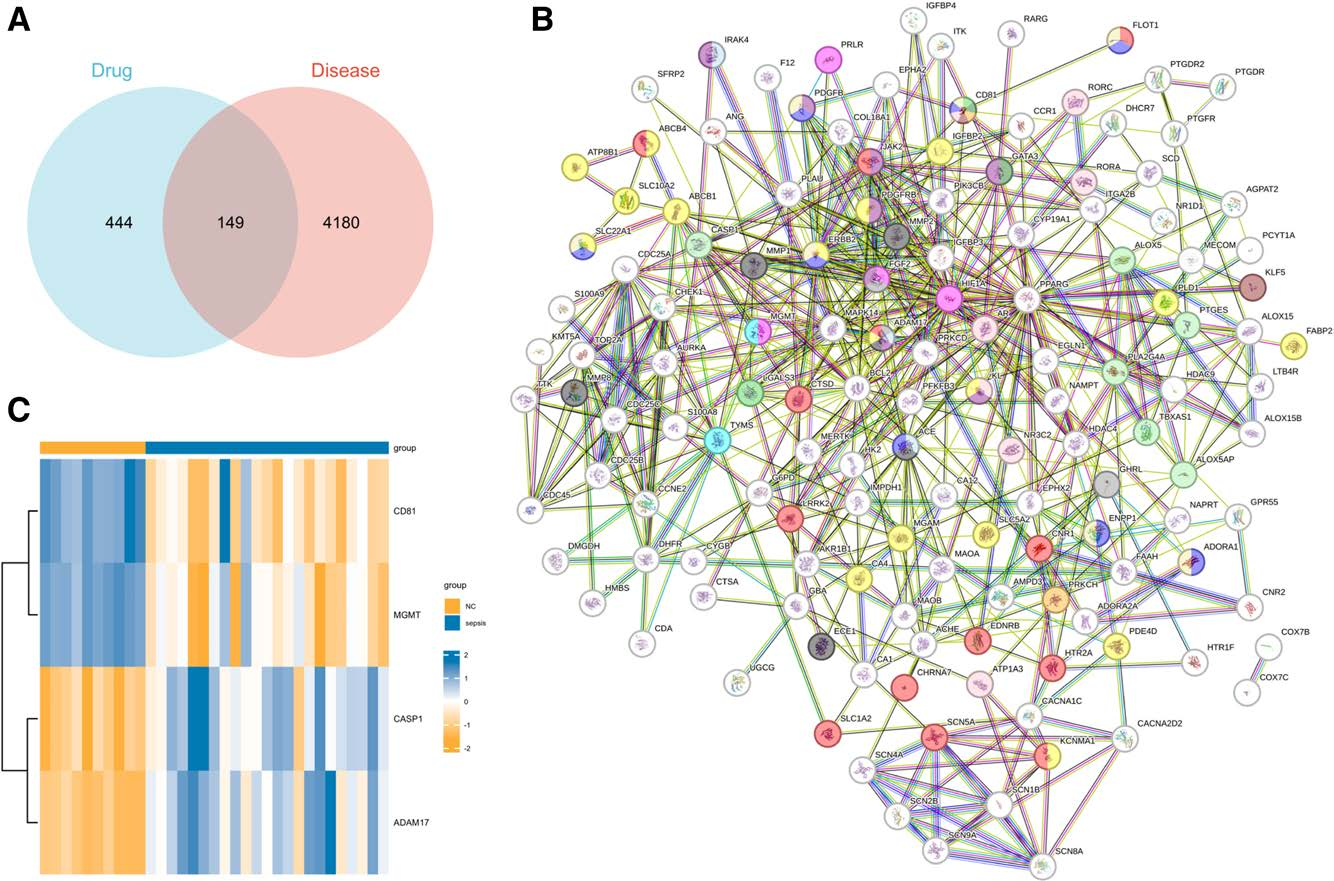
Acquisition and Analysis of Intersecting Genes. A. The blue section represents 593 drug targets, the pink section represents 4329 disease targets, and the central area consists of 149 intersecting genes. B. The network of PPIs, with ADAM17, CASP1, CD81, and MGMT at the center. C. Heatmap of the 4 core genes in the PPI network center (ADAM17, CASP1, CD81, and MGMT). In the representation, blue signifies elevated expression levels, whereas yellow represents reduced expression levels.

Twenty active components of Calculus Bovis were obtained from the HERB database. Through the SwissTargetPrediction database, 373 potential targets were identified, and 11 active components along with 337 potential targets were derived from the BATMAN-TCM database. After intersecting and deduplicating, 29 active components and 593 potential targets were finalized. Subsequently, To illustrate the relationship between putative Calculus Bovis targets and differentially expressed genes in sepsis, a Venn diagram was made. Yielding 149 intersecting genes (Fig. 3A). Finally, a table of intersecting genes with corresponding active components and their properties was compiled (Table 2).

**Table 2:** Active ingredients and target of calculus bovis

ingredients	Ingredient id	Molecule weight	Target
2-[(3alpha,12alpha-dihydroxy-24-oxo-5beta-cholan-24-yl)amino]ethanesulfonic acid	HBIN003943	499.7	CA1
amygdalin	HBIN015934	457.43	MGMT CDA MMP8
Belamcandol A	HBIN017716	362.55	CNR1 ALOX5
chenodeoxycholic acid	HBIN020280	392.57	MMP2 PTGES
cholesterol	HBIN020400	386.65	PRKCD CCR1
CLR	HBIN021150	386.65	RORA
Deoxycholic Acid	HBIN023356	392.57	EDNRB
ergosterol	HBIN025553	396.65	KL CYP19A1
GCH	HBIN027403	465.62	CTSA CNR1
irigenin	HBIN030301	360.31	ADORA1 MAOA
Irisflorentin	HBIN030311	386.35	ALOX5AP PLAU
isoiridogermanal	HBIN030826	474.72	JAK2 PRKCH
mangiferin	HBIN034394	422.34	CA12 AKR1B1
Mangiferolic acid	HBIN034395	456.7	SLC10A2 AR
Methyl desoxycholate	HBIN035169	406.6	CA1
N-(3alpha,12alpha-dihydroxy-5beta-cholan-24-oyl)glycine	HBIN036231	449.62	CTSA LTB4R
oleanolic acid	HBIN037940	456.7	CES2
tectoridin	HBIN045832	462.4	MMP2 CA12
tectorigenin	HBIN045833	300.26	PLAU IGFBP3
ursolic acid	HBIN047613	456.7	RORA PRKCH
Deoxycholicacid	HBIN023356	392.57	PTGDR AR
Choline	HBIN020406	104.17	DMGDH ATP8B1
Deoxycorticosterone	HBIN023358	330.5	NR3C2 F12
Tyr-Gly	HBIN047438	238.24	OPRD1
Ergotamine	HBIN025558	581.7	ADAM17 CYGB
Bilirubin	HBIN018510	584.7	DHFR
Lithospermate B	HBIN033408	740.9	IMPDH1
Biliverdin	HBIN018511	580.6	SFRP2NAMPT
Cholicacid	HBIN020404	408.6	ADORA1 PTGDR

### 3.3. PPI analysis

After excluding irrelevant data, the improved PPI network included 140 nodes. Genes like ADAM17, CASP1, CD81, and MGMT were located at the center of this network (Fig. 3B). These genes are associated with the activation of T cells, neutrophil-mediated immunity, mammary epithelial cell differentiation, and positive regulation of cellular receptor signaling pathways. This indicates their potential significance as targets for Calculus Bovis in improving the prognosis of sepsis.

### 3.4. Construction of the “Drug–Active Component–Target” network

**Figure 4.**
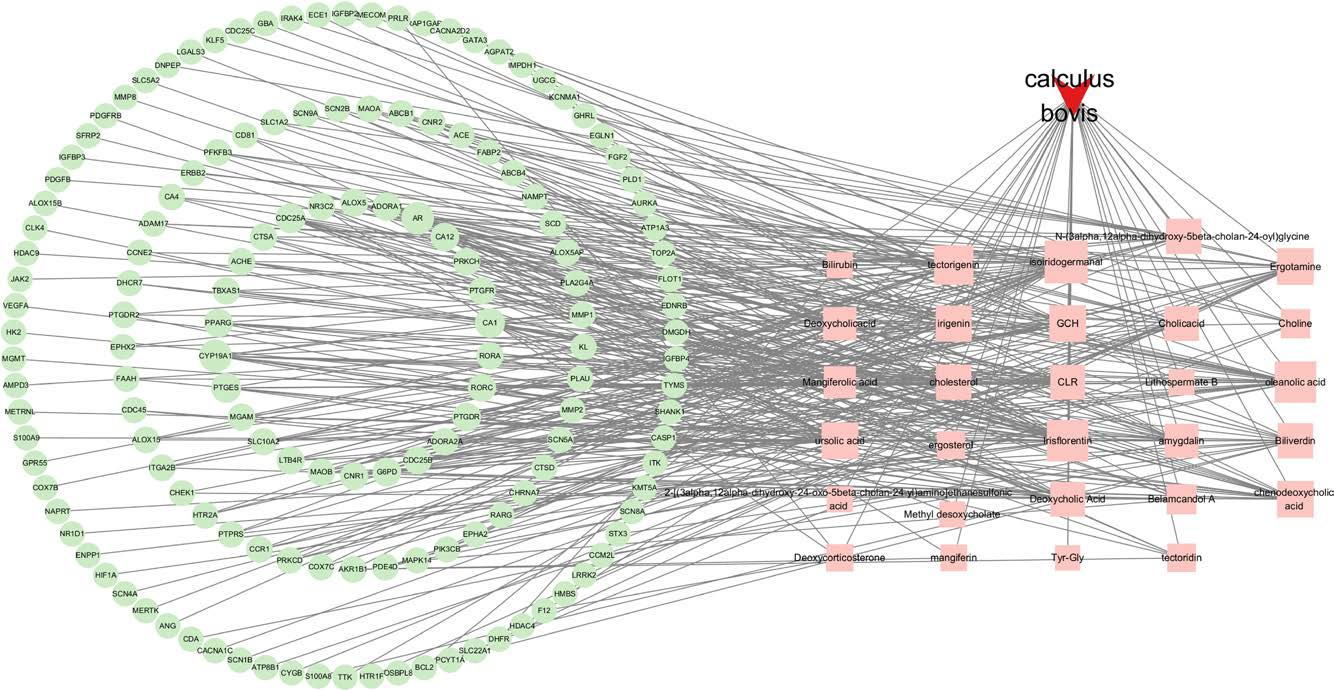
Drug Component Target Network Diagram. In this network diagram, genes that intersect are depicted as circular nodes, active ingredients of Calculus Bovis are shown as square nodes, and the inverted triangular nodes symbolize Calculus Bovis itself. The connecting lines illustrate the interactions among the drug, its compounds, and their targets.

Using Cytoscape version 3.10.1, a network comprising 149 intersecting targets and drug components was established. Within this network, the circular nodes symbolize the intersecting targets, the square nodes denote the active constituents of the drug, and the inverted triangular nodes represent Calculus Bovis. Lines indicate the interactions between components and targets. Network Analyzer plugin was used for degree analysis. The larger the degree, the larger the network node. Among the intersecting target genes, AR and CYP19A1 exhibited the highest degree values. Among the active compounds, isoiridogermanal displayed the highest degree, suggesting their potential key roles in the treatment of sepsis (Fig. 4).

### 3.5. GO and KEGG enrichment analysis

**Figure 5.**
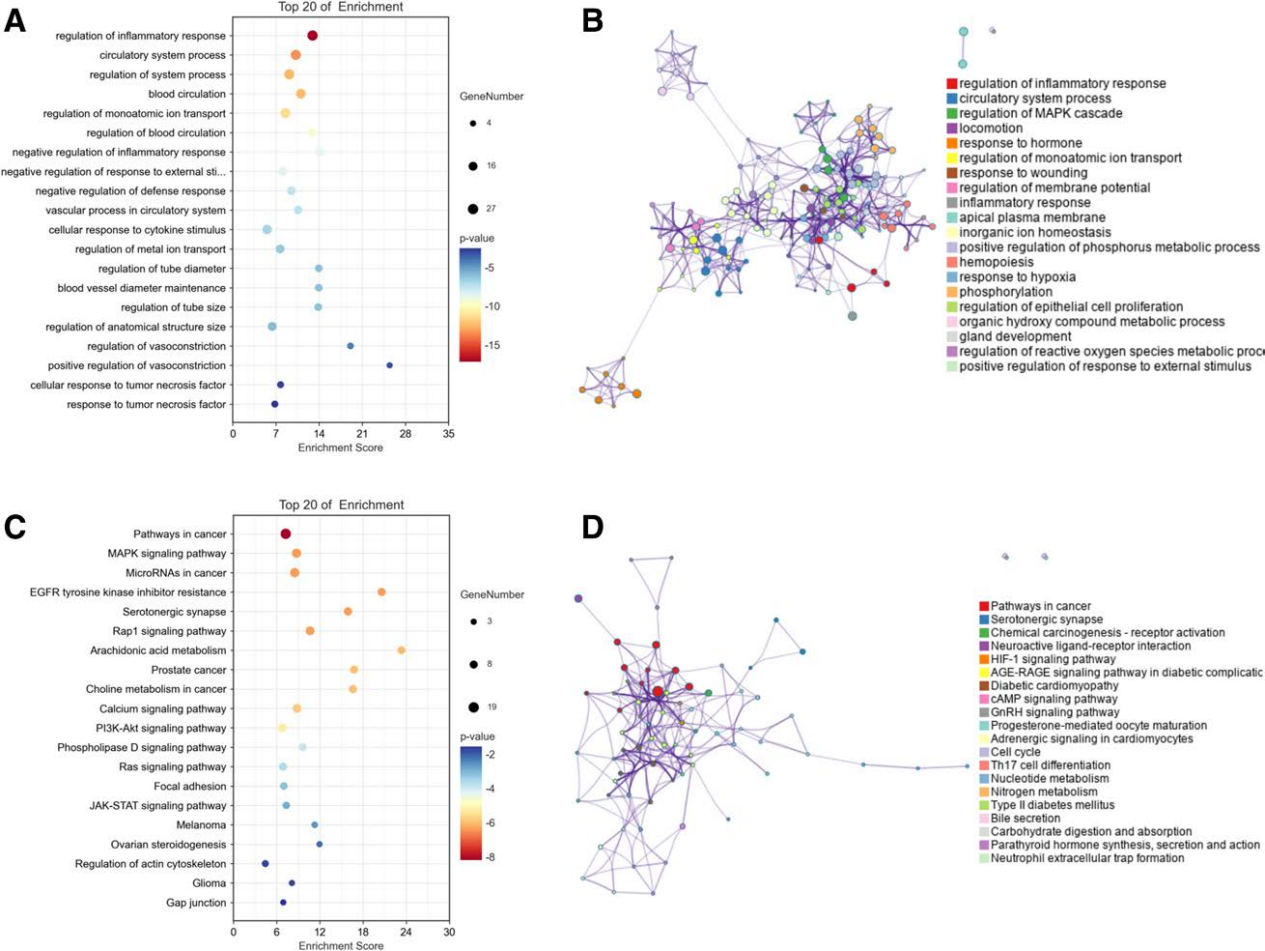
GO and KEGG Enrichment Analysis of Intersecting Targets. A. The size of the bubbles denotes the quantity of genes involved, while the depth of the color represents the significance of the *P* value. The genes that intersect predominantly participate in the modulation of inflammatory responses and various processes related to the circulatory system, along with other biological functions. B. Each node is colored differently, corresponding to different biological processes (Cluster ID). C. In this representation, the bubble’s size corresponds to the count of genes, while the intensity of the color signifies the *P* value. The genes at the intersection are chiefly engaged in diverse signaling pathways, including those related to cancer, immune synapses, and receptor activation in chemical carcinogenesis. D. Each node is colored differently, corresponding to different signaling pathways (Cluster ID).

Based on the GO annotations of intersecting targets, a comprehensive compilation of 5970 gene functions was obtained. In this collection, 4723 were enhanced in biological processes (BP), 408 in cellular components (CC), and 839 in molecular functions (MF). GO enrichment analysis revealed that the targets were mainly involved in inflammatory responses, cell pyroptosis, and inflammatory reactions, among others (Fig. 5A). KEGG pathway analysis identified that target genes are mainly involved in signaling pathways such as cancer pathways, immune synapses, receptor aggregation, and signal transduction (Fig. 5C). To further elucidate their interconnections, we display herein the findings from the GO and KEGG enrichment analyses using a network diagram. In this diagram, terms sharing a similarity greater than 0.3 are linked by lines. The most statistically significant term from each of the 20 clusters was chosen, adhering to a limit of no more than 15 terms per cluster and a maximum of 250 terms in total. This network was depicted using the Cytoscape software, where each node symbolizes a term and is initially color-coded based on its cluster identification (Fig. 5B and D).

### 3.6. Survival curve analysis

**Figure 6.**
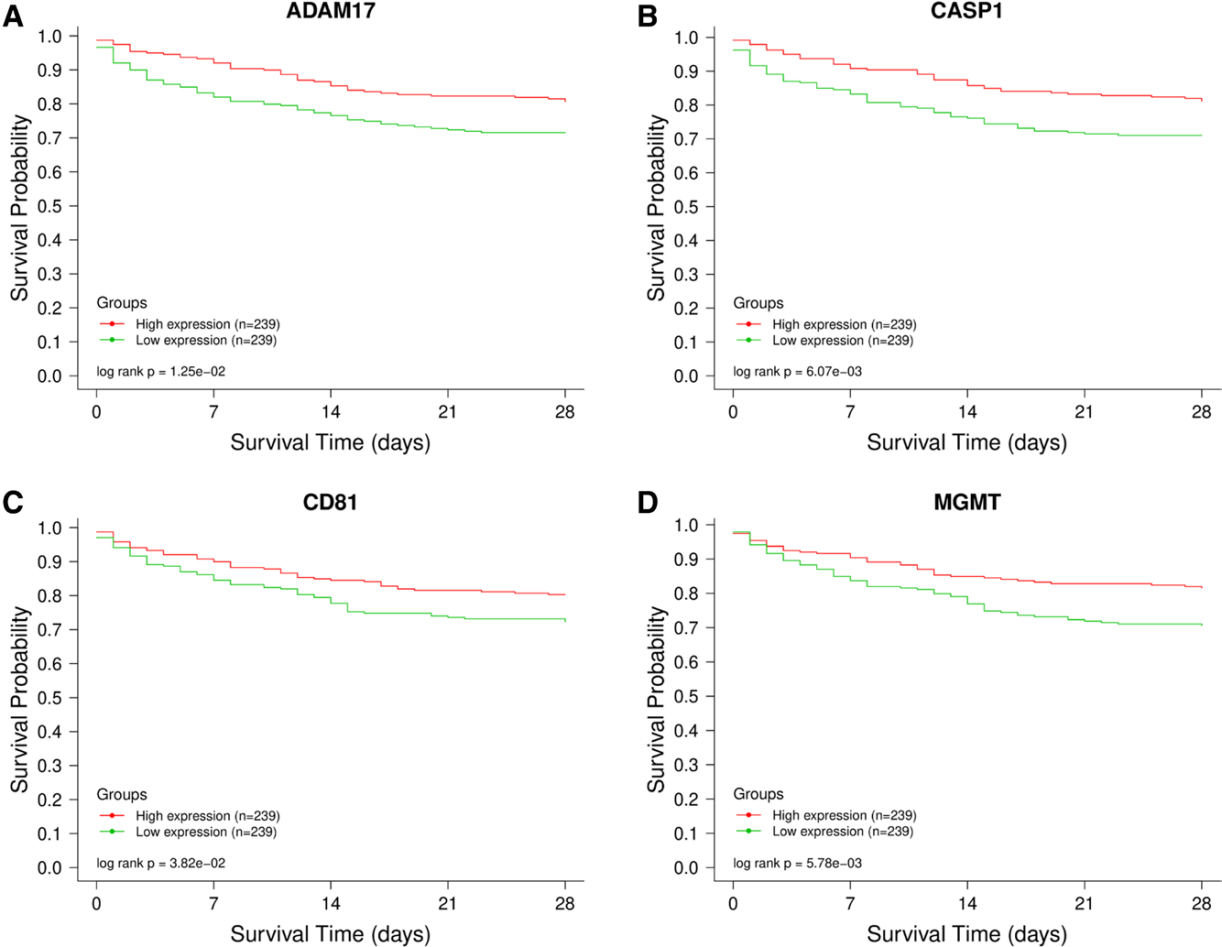
Illustrates the analysis of survival curves for the 4 critical targets. On the graph, the horizontal axis denotes the duration of survival in days, while the vertical axis indicates the rate of survival. The green line depicts samples with low mRNA expression stability index (mRNA), and the red line portrays samples with high mRNA (*P* < .05).

**Figure 7.**
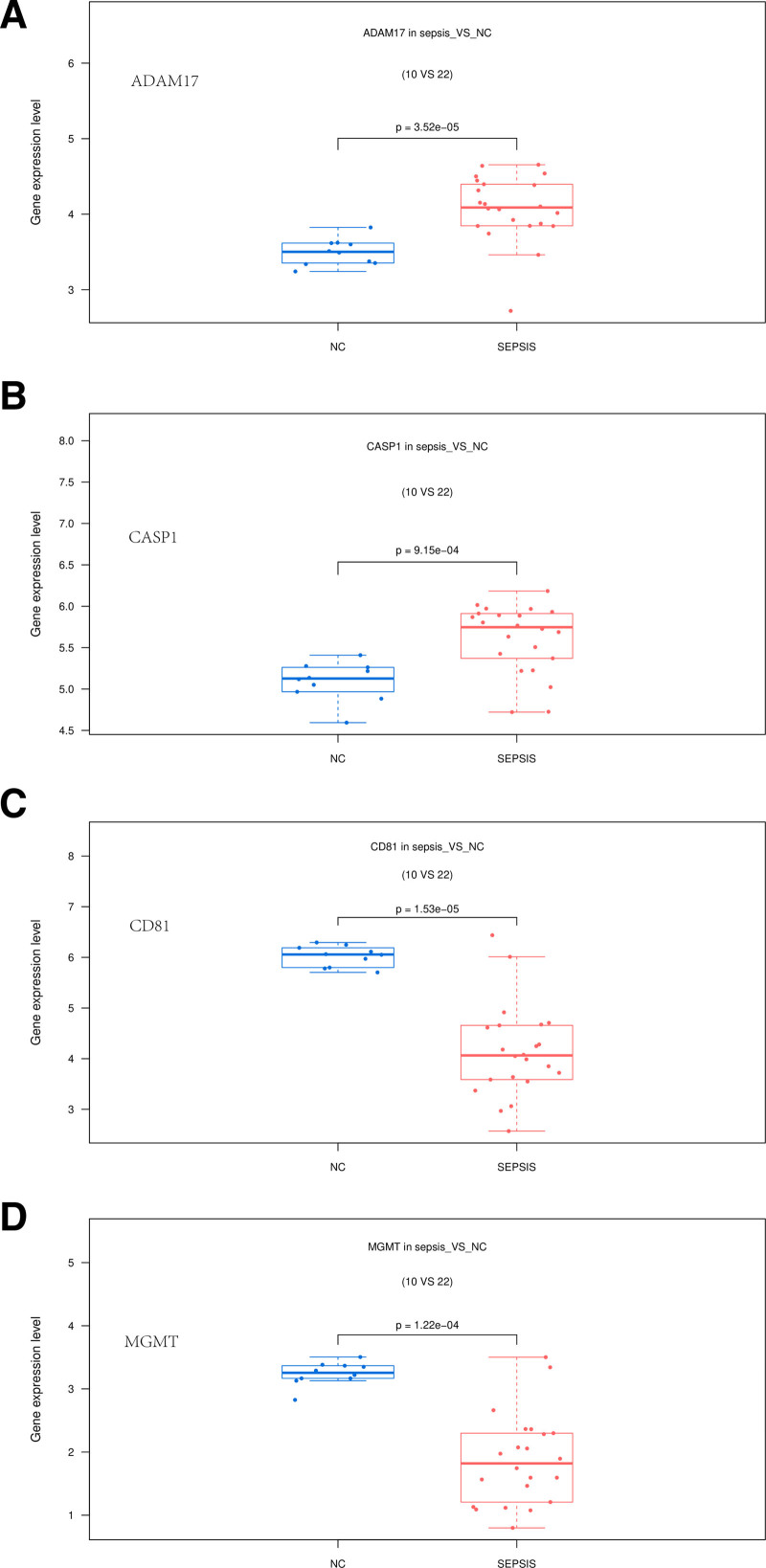
Presents the expression patterns of the 4 pivotal genes. As evident from the figure, ADAM17, and CASP1 exhibited elevated expression in the sepsis group. Conversely, CD81 and MGMT demonstrated reduced expression in the same group (*P* < .05).

To assess the correlation between patient prognosis and the 149 intersecting targets, we conducted a prognostic analysis using the public database GSE65682.^[13]^ Through log-rank tests, the expression of 4 genes, namely, ADAM17, CASP1, CD81, and MGMT, was found to be associated with prognosis (Fig. 6A–D). The greater the gap between the 2 curves, the more pronounced the change in patient prognosis. In the high-expression groups of ADAM17, CASP1, CD81, and MGMT, the survival rate was significantly higher, indicating a better prognosis. Hence, ADAM17, CASP1, CD81, and MGMT are likely to be potential core targets in Calculus Bovis treatment for sepsis. Box plots reflect the expression levels of these 4 key genes in normal and sepsis groups (Figs. 7A–D).

### 3.7. Construction of the diagnostic model

**Figure 8.**
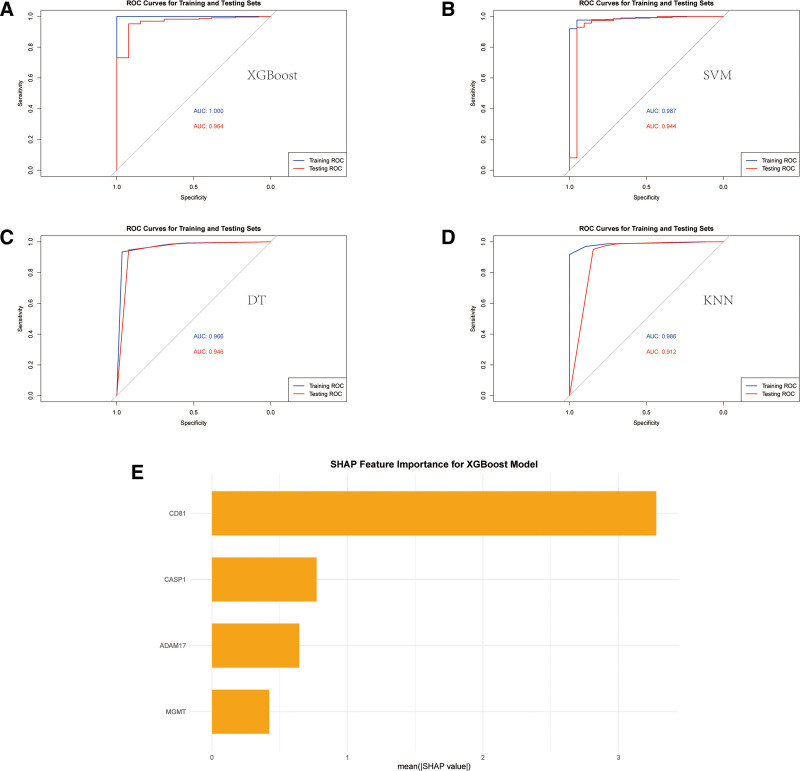
ROC curves of sepsis diagnostic models constructed using 4 machine learning algorithms (XGBoost, SVM, Decision Tree, KNN) and SHAP feature importance analysis of XGBoost model. A–D: shows the ROC curves of XGBoost (A), SVM (B), Decision Tree (C), and KNN (D) models on both the training set and the test set. The blue curves represent the ROC curves on the training set, and the red curves represent the ROC curves on the test set. The AUC values are shown in each graph, indicating the classification performance of the model. The AUC values of the XGBoost model on the training set and test set are 1.000 and 0.964, respectively, which shows a very high prediction accuracy. E: Shows the SHAP feature significance analysis of XGBoost model Fig. CD81 has the highest SHAP value, indicating that it contributes the most to the prediction of sepsis diagnosis model, followed by CASP1, ADAM17, and MGMT.

The diagnostic prediction of sepsis was performed by the models constructed by the 4 machine learning algorithms described above using the GSE65682 dataset. The model performance was evaluated by ROC curves (Figs. 8A–D). The AUC values of the XGBoost model in the training and test sets were 1.000 and 0.964, respectively, which showed very high prediction accuracies. The AUC values of the SVM model in the test set were 0.944, the Decision Tree was 0.946, and the KNN model had an AUC value of 0.912, which all showed a good classification effect. In addition, the analysis of SHAP values based on the XGBoost model (Fig. 8E) revealed the importance of 4 genes, CD81, CASP1, ADAM17, and MGMT, in the diagnostic model. cD81 contributed the most, suggesting that its potential role in sepsis diagnosis is the most significant. These results demonstrate the significant application of the model constructed using machine learning algorithms in the early diagnosis of sepsis.

### 3.8. Immunofluorescence analysis

**Figure 9.**
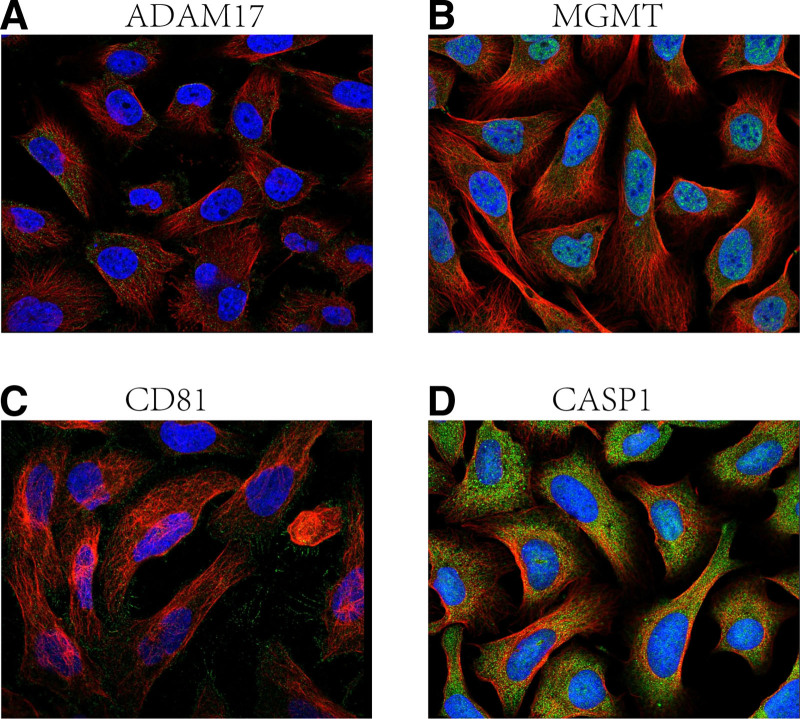
Protein localization of 4 genes, ADAM17, MGMT, CD81, and CASP1, demonstrated by immunofluorescence analysis. A-D: Immunofluorescence staining of 4 genes, ADAM17 (A), MGMT (B), CD81 (C), and CASP1 (D), inside the cell is shown. Green fluorescence represents target proteins, red fluorescence labels microtubules, and blue is staining of the nucleus. ADAM17 and CD81 are mainly localized in the cell membrane, while MGMT is mainly distributed in the nucleus, and CASP1 is shown to be distributed as cytoplasmic lysate.

**Figure 10.**
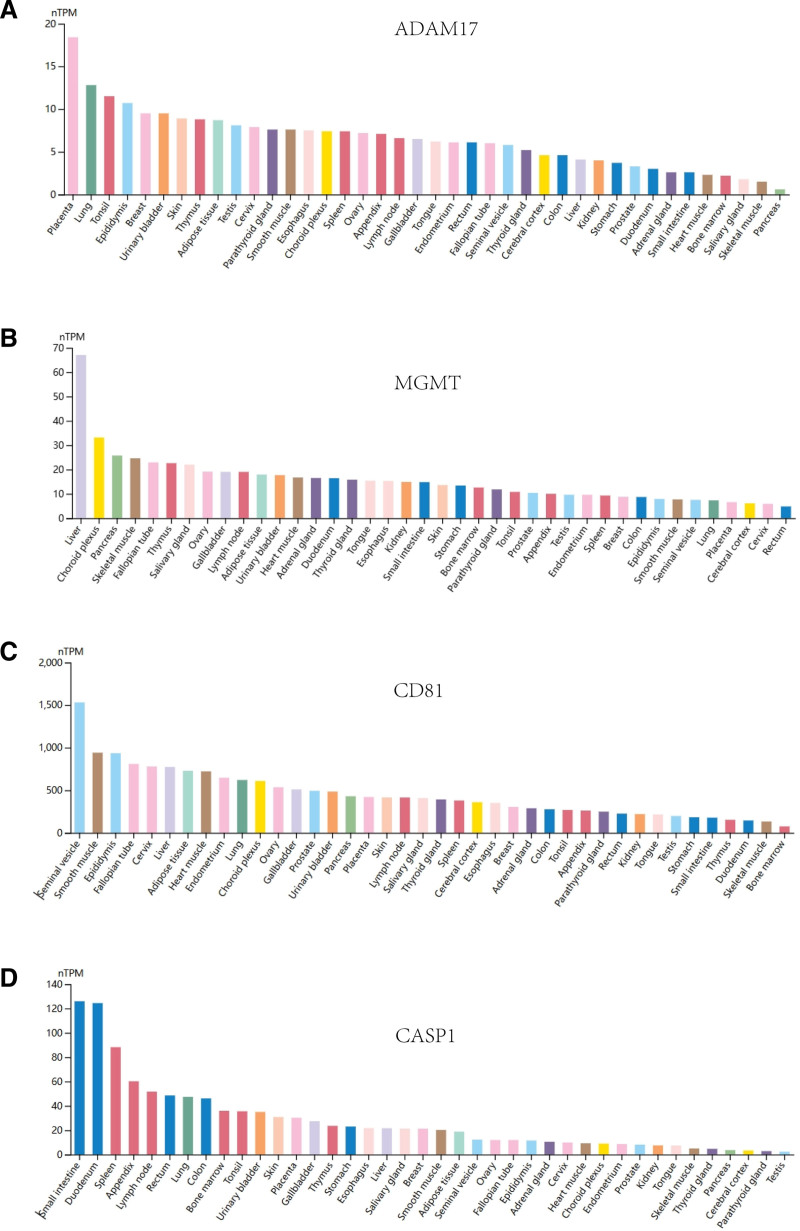
Demonstrates the relative expression levels of the 4 genes in different human tissues. A–D: ADAM17 is highly expressed in the placenta and lungs, MGMT has the highest expression in the liver and ovaries, CD81 is highly expressed in kidney and muscle tissues, while CASP1 shows high expression levels in the small intestine and colon.

Immunofluorescence analysis showed that ADAM17 (Fig. 9A) was mainly localized in the cell membrane and cytoplasm, suggesting that it may play an important role in cell surface and intracellular signaling.MGMT (Fig. 9B) was predominantly distributed in the nucleus of the cells, which is consistent with its known DNA repair function.CD81 (Fig. 9C) was mainly localized in the cell membrane, suggesting that it may be involved in cell-to-cell signaling or immune regulation. CASP1 (Fig. 9D) was shown to be distributed in the cytoplasmic lysate, which may be related to its function in regulating apoptosis. Tissue-specific expression analysis further revealed differences in the expression of the 4 genes in different tissues. adam17 showed high expression in placental and lung tissues (Fig. 10A), whereas MGMT showed the highest expression levels in the liver and ovary (Fig. 10B). cd81 showed strong expression in kidney and skeletal muscle (Fig. 10C), and CASP1 was highly expressed (Fig. 10D). These data suggest that these genes exert different biological functions in different tissues and provide clues to their potential roles in sepsis. The analysis of immunofluorescence images and tissue-specific expression data revealed the different expression patterns of ADAM17, MGMT, CD81, and CASP1 at the cellular level and tissue level, which lays the foundation for further investigation of their roles in sepsis.

### 3.9. Single-Cell RNA Sequencing

**Figure 11.**
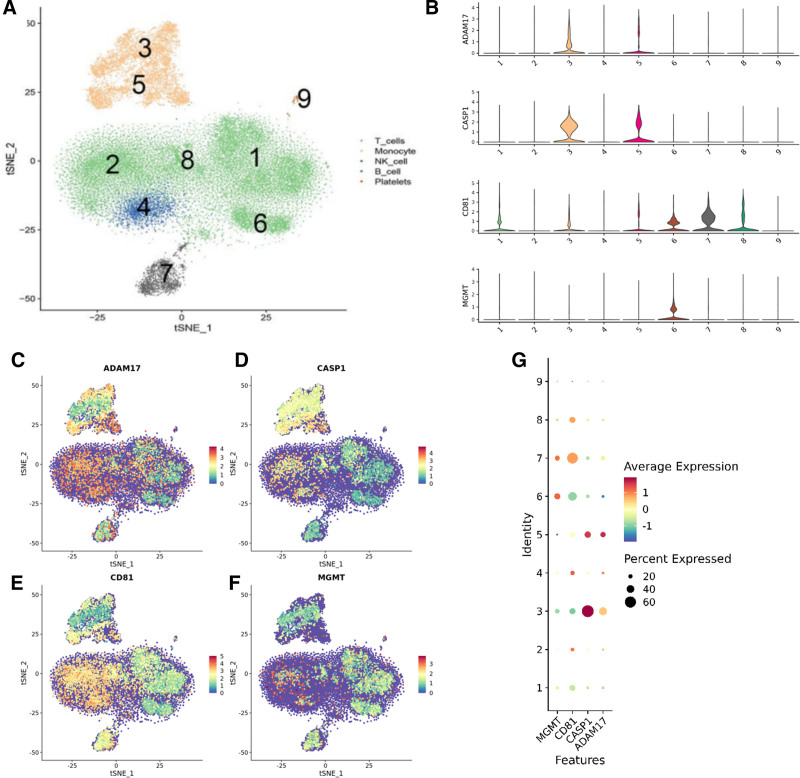
Analysis of single-cell sequencing results. A. Macrophages were present in groups 3 and 5, natural killer cells were present in group 4, and T lymphocytes were represented by groups 1, 2, 6, and 8. Group 7 represents B cells, and group 9 represents platelets. B–F. CASP1 is primarily located in group 3, the macrophage lineage, while MGMT, ADAM17, and CD81 are widely distributed among various immune cells. G. The bubble chart illustrates the relative abundance and the distribution percentages of each central gene expressed across various cell populations.

Based on the transcriptomic sequencing analysis of the aforementioned 5 cell samples (Fig. 11A), the 4 key genes identified in this study–ADAM17, CASP1, CD81, and MGMT–were submitted to the single-cell library to determine cell lineage localization. CASP1 was found to be primarily located in group 3, the macrophage lineage, while MGMT, ADAM17, and CD81 were widely distributed across various immune cells (Fig. 11B–G).

### 3.10. Molecular docking

**Figure 12.**
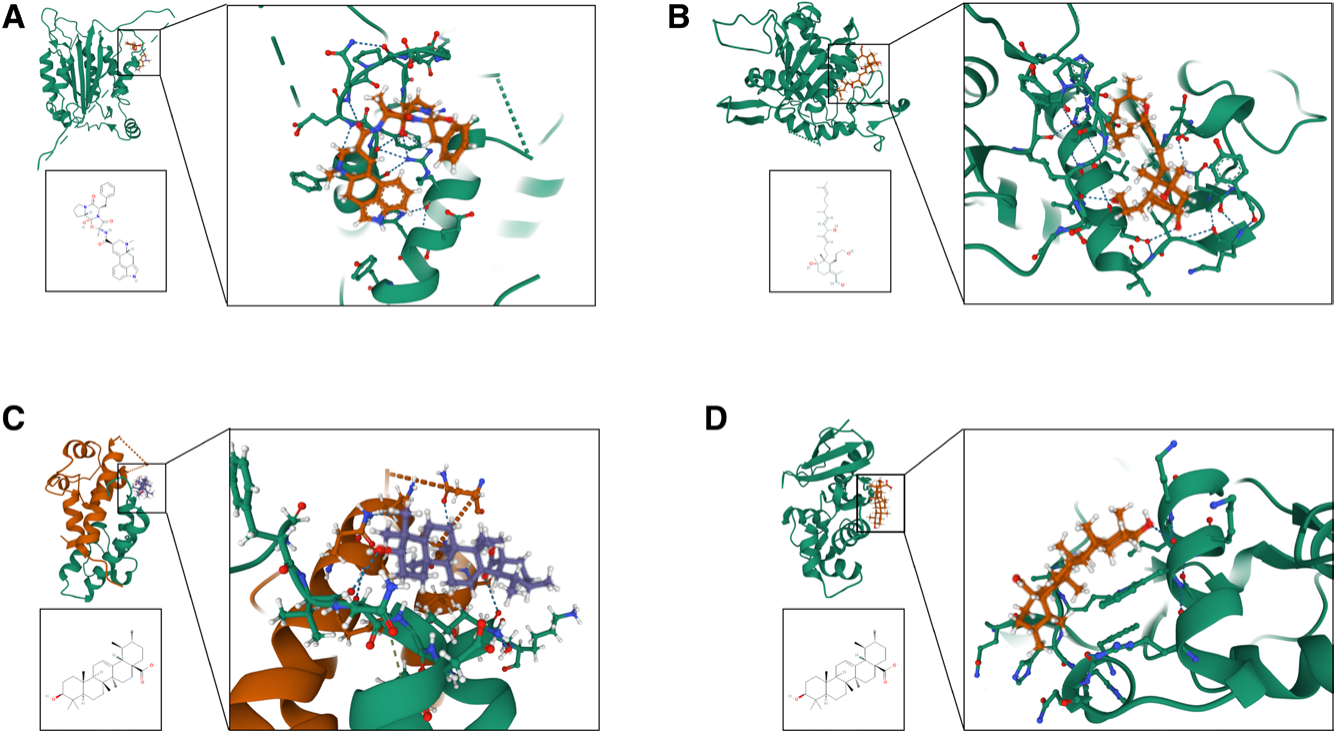
Molecular Docking Results. A. The binding affinity of isoiridogermanal with ADAM17 is −6.004 kcal/mol. B. The binding affinity of ergotamine with CASP1 is −7.203 kcal/mol. C. The binding affinity of ursolic acid with CD81 is −7.952 kcal/mol. D. The binding affinity of ursolic acid with MGMT is −8.044 kcal/mol.

Based on the aforementioned analysis, we conducted molecular docking of Calculus Bovis active components, isoiridogermanal, ergotamine, and ursolic acid, with ADAM17 (Fig. 12A), CASP1 (Fig. 12B), CD81 (Fig. 12C), and MGMT (Fig. 12D). A binding affinity value below −4.25 kcal/mol suggests active binding between the ligand and its target, a value below −6.0 kcal/mol denotes robust binding activity, and a value below −7.0 kcal/mol signals substantial docking interactions.^[25]^ The results of docking these active components with their respective targets are presented in Table 3, with ursolic acid showing the best docking activity with MGMT.

**Table 3:** Molecular docking results

Compounds	Targets	Bind energy
isoiridogermanal	ADAM17	−6.004 kcal · mol^−1^
Ergotamine	CASP1	−7.203 kcal · mol^−1^
ursolic acid	CD81	−7.952 kcal · mol^−1^
ursolic acid	MGMT	−8.044 kcal · mol^−1^

### 3.11. Molecular dynamics simulation

**Figure 13.**
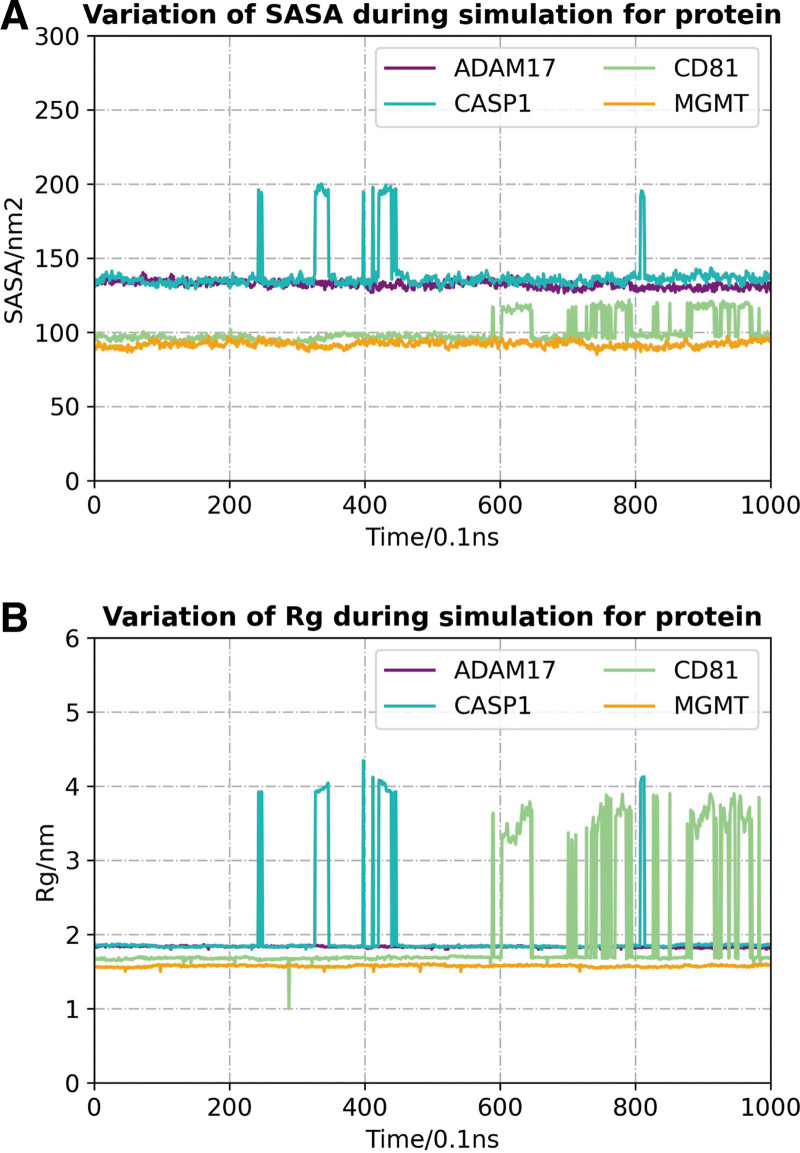
SASA, Rg analysis. A: The trend of solvent-accessible surface area (SASA) showed that CASP1 and CD81 fluctuated during the simulation, suggesting that these proteins had a large change in conformation during the simulation. B: The change of radius of gyration (Rg) showed that the overall conformation of CASP1 and CD81 was not as stable as that of ADAM17 and MGMT.

**Figure 14.**
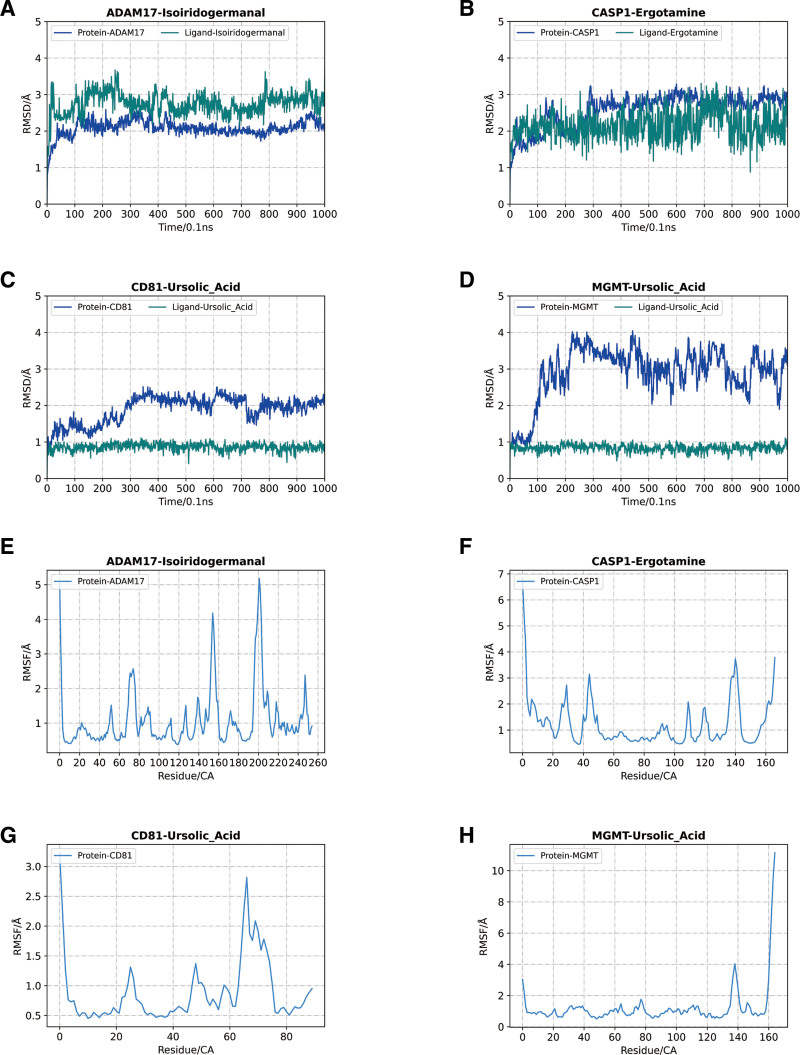
RMSD and RMSF analyses. A–D: RMSD analyses demonstrate the stability of each protein-small molecule complex during the simulation.ADAM17-Isoirdogermanal and CD81-Ursolic Acid complexes show high stability, while CASP1-Ergotamine and MGMT- Ursolic Acid complexes showed large fluctuations. E–H: RMSF analysis revealed the fluctuations of the proteins at the residue level. The binding site regions of ADAM17 and CD81 showed small fluctuations, whereas those of CASP1 and MGMT showed large fluctuations, suggesting that the conformational changes in these regions are more significant.

The results of MD showed that the solvent-accessible surface area (SASA) of the proteins changed relatively little during the 100 ns MD, demonstrating overall stability. However, CASP1 and CD81 exhibited slight fluctuations, especially around 600 ns and 800 ns, suggesting that these 2 proteins had some conformational changes during the simulation (Fig. 13A). The results of the radius of gyration (Rg) analysis further supported this conclusion, with the Rg values of CASP1 and CD81 fluctuating within the same time period, suggesting that the overall conformation of these 2 proteins is more unstable than the other proteins (Fig. 13B). In the RMSD analysis of each protein-small molecule complex, ADAM17-Isoirdogermanal complex (Fig. 14A) and CD81-Ursolic Acid complex (Fig. 14B) showed relatively stable RMSD values, suggesting that these complexes maintained a more stable binding state during the simulation. In contrast, the RMSD values of CASP1-Ergotamine (Fig. 14C) and MGMT-Ursolic Acid (Fig. 14D) complexes showed large fluctuations at the later stage, suggesting that they may have lost part of their stability over a longer period of time. The residue-level fluctuations of the proteins were visualized by RMSF analysis (Fig. 14E–H). The key binding site regions of ADAM17 and CD81 showed less fluctuation, suggesting that these regions maintained better stability during the simulation. In contrast, the binding site regions of CASP1 and MGMT fluctuated more, indicating that the conformation of these regions changed considerably during the simulation process, which may affect their binding stability with small molecules. Taken together, ADAM17 and CD81 showed more stable binding to small molecules, whereas the CASP1 and MGMT complexes showed larger fluctuations, suggesting poorer binding stability. These results provide theoretical support for the design of small-molecule drugs targeting these proteins.

## 4. Discussion

Sepsis ranks among the prevalent complications in patients with critical illnesses and is a leading cause of mortality globally, accounting for nearly 50 million cases and 11 million deaths annually.^[26]^ The dysfunction of organs and increased mortality associated with sepsis are often ascribed to the intricate interaction of the initial inflammatory response and the later counteracting anti-inflammatory reactions.^[27]^ Present-day therapeutic strategies for sepsis predominantly concentrate on managing the inflammatory response and mitigating damage to organ functions. These strategies encompass anti-infection protocols, hormone therapy, mechanical breathing support, and providing nutritional aid.^[28]^ Nonetheless, there remains a need for more specific and efficacious treatments. TCM contributes notably to sepsis treatment by suppressing inflammatory responses, diminishing oxidative stress, bolstering immunity, and preserving cellular equilibrium.^[29]^ As a result, this research pinpointed potential targets for Calculus Bovis in sepsis therapy using network pharmacology approaches and clinical prognostic data. These targets, including ADAM17, CD81, CASP1, and MGMT, lay the foundation for further research into the mechanisms of action of Calculus Bovis in sepsis treatment.

O^6^-methylguanineethylguanine-DNA methyltransferase (MGMT) is an enzyme involved in DNA repair.^[30]^ MGMT regulation is governed by epigenetic mechanisms, and the methylation state of its promoter region is linked to both the development and the prognostic outcomes of glioblastoma.^[31]^ Our survival curve analysis revealed that the higher expression of MGMT in groups with a better prognosis of sepsis led to increased survival rates. The increased expression of MGMT might be beneficial in sepsis. Moreover, RNA-seq analysis identified a broad distribution of this gene across different immune cell types. Analysis through network pharmacology indicated that an active constituent of Calculus Bovis, ursolic acid, targets MGMT and exhibits antibacterial properties.

The CASP1 gene encodes caspase-1, which plays a significant role in inflammatory processes.^[32]^ CASP1 is a well-known gene involved in pyroptosis, and metabolites of benzene have been found to trigger cell death through the activation of the Aim2/CASP1 pathway.^[33]^ It has been established that cell pyroptosis significantly contributes to the activation of hemostasis in the context of sepsis.^[34]^ Survival curve analysis revealed that increased expression of CASP1 in groups with better sepsis prognosis led to higher survival rates. CASP1 upregulation might be advantageous in sepsis, and our RNA-seq analysis revealed that CASP1 was primarily located in the macrophage lineage. Network pharmacological analysis indicated that the active components of Calculus Bovis and ergotamine targeted CASP1 and exhibited antibacterial properties.

ADAM17 acts as a crucial proteolytic sheddase, playing a pivotal role in inflammatory responses, particularly in the context of sepsis.^[35]^ The shedding of the extracellular domain of ADAM17 can improve neutrophil recruitment, thereby enhancing neutrophil function during sepsis and boosting the body’s immunity.^[36]^ Our survival curve analysis demonstrated that increased expression of ADAM17 in groups with a better prognosis of sepsis resulted in higher survival rates. ADAM17 upregulation might be beneficial in sepsis, and our RNA-seq analysis showed that ADAM17 was extensively dispersed among various immune cell kinds. Our network pharmacological analysis showed that the therapeutic component of Calculus Bovis, isoiridogermanal, targeted ADAM17 and exhibited antibacterial properties.

CD81 participates in various crucial cellular processes. Within the immune system, CD81 is instrumental in regulating immune synapses, the clustering of receptors, and the process of signal transduction.^[37]^ Additionally, as a member of the tetraspanin integral membrane protein family, CD81 has been identified as an essential receptor for HCV (hepatitis C virus).^[38]^ Our survival curve analysis revealed that increased expression of CD81 in groups with a better prognosis of sepsis led to higher survival rates. CD81 upregulation might be beneficial in sepsis. Furthermore, our RNA-seq analysis suggested that CD81 was extensively present across diverse types of immune cells. Analysis using network pharmacology highlighted that an active ingredient of Calculus Bovis, ursolic acid, targeted CD81 and exhibited antibacterial properties.

This study has a few limitations, which are addressed below:

1) By predicting the targets of TCM compounds, we have delineated the potential targets and pathways of these components, establishing their connection with disease processes. However, this method of component – target pathway – disease research is more applicable to single-component chemical drugs and may not fully apply to traditional Chinese medicine with multiple components and multi-target synergistic effects.
2) Further cellular experiments are required to determine whether small molecules exert an inhibitory or promotive effect on key targets.
3) To further clarify the pharmacological action of Calculus Bovis, clinical trials and evidence-based medical studies are needed.

## Author contributions

**Data curation:** Hao Wang.

**Investigation:** Yingchun Hu.

**Writing – original draft:** Li Hu.
